# Kidney Cancer: Current Progress in Treatment

**DOI:** 10.4021/wjon345w

**Published:** 2011-08-24

**Authors:** Ankita Thakur, Sunil K. Jain

**Affiliations:** a Faculty of Pharmacy, Adina Institute of Pharmaceutical Sciences, Sagar, M.P. - 470002, India

**Keywords:** Kidney cancer, Immunotherapy, Sunitinib, Sorafenib, Nephrectomy

## Abstract

Kidney cancer accounts for approximately 2% of all cancers worldwide, with renal cell carcinoma being the most widespread form. Worldwide, the occurrence and mortality rates are rising by 2–3% per decade. Cigarette smoking, obesity, acquired cystic kidney disease and inherited vulnerability are identified risk issues for kidney cancer. Immunotherapy confers a small but significant overall survival benefit in metastatic renal cell carcinoma but only for the minority of patients, i.e. the 20% with good predictive characteristics. Current developments in the molecular biology of renal cell carcinoma have recognized multiple pathways related with the progress of this cancer. Several strategies have been explored targeting these trails, with major clinical benefits shown in early studies. New agents including the small molecule targeted inhibitors like sunitinib, sorafenib and temsirolimus, and the monoclonal antibody bevacizumab have shown anti-tumour activity in randomised clinical trials and have become the standard of care for most patients.

## Introduction

Kidney cancer includes renal cell carcinoma (cancer that forms in the lining of very small tubes in the kidney that filter the blood and remove waste products) and renal pelvis carcinoma (cancer that forms in the centre of the kidney where urine collects). Kidney cancer is the seventh most common cancer and the tenth most common cause of cancer death for men. It is the eighth most common cause of cancer for women [1]. In 2010, an estimated 58,240 adults (35,370 men and 22,870 women) in the United States will be diagnosed with kidney cancer and renal pelvic cancer [1]. It is estimated that 13,040 deaths (8,210 men and 4,830 women) from this disease will occur this year. The five-year relative survival rate (percentage of people who survive at least five years after the cancer is detected, excluding those who die from other diseases) of people with kidney cancer is about 68% [1]. Early stage detection can make the disease curable but metastatic kidney cancer is usually lethal. Kidney cancer tends to be “silent,” causing no symptoms until it has spread outside the kidneys. The most common form of kidney cancer in children is Wilms’ tumor, which also reveals distinctive genetic abnormalities and biologic activities.

Renal cell carcinomas (RCC) are classified histologically as clear cell (60 - 80%), papillary (10 - 15%), chromophobe (5 - 10%) and collecting duct (< 1%). Clear-cell histology is related with an improved product more than papillary or chromophobe histology in the metastatic setting [3] however the reverse is true for localised disease [2, 3]. Furthermore, RCC has a poor prediction and is extremely resistant to treatment with chemotherapy [4]. Targeted drugs are becoming the recommended first- and second-line treatment alternatives for patients with RCC [5]. Because of their selectivity, targeted agents are normally better tolerated than standard cytotoxic treatments [6]. Results from clinical studies have shown positive efficiency in RCC subsequent treatment with targeted therapy such as sunitinib [7], sorafenib [8], temsirolimus [9], and the combination of bevacizumab with interferon a [10, 11]; all of these agents have been accepted for use in RCC. Everolimus, an oral mammalian target of rapamycin inhibitor, has also revealed activity in the treatment of RCC, particularly in patients who developed on earlier treatment with vascular endothelial growth factor (VEGF)–targeted therapy [12].

The initial part of present review mainly highlights general idea regarding cause, risks, and symptoms of kidney cancer. However, latter part focuses on the brief introduction about the efforts that have been taken for combating kidney cancer including various surgical and non surgical therapies. The new and emerging drugs and the combination therapy are found to be promising to defeat the kidney cancer.

## Types of Kidney Cancer [13]

On the basis of microscopic view the renal cell cancer (RCC) is having following subtypes:

### Clear cell renal cell carcinoma

This is the most widespread form of RCC. About 7 out of 10 people with RCC have this sort of cancer. Under microscopic observation, the cells that make up clear cell RCC appear very pale or clear.

### Papillary renal cell carcinoma

This is the second most common subtype and about 1 out of 10 people with RCC have this type. These cancers make tiny finger-like projections (called papillae) in some, if not most, of the tumor. As per some physicians these are also entitled as cancers chromophilic for the reason that the cells acquire certain dyes used to organize the tissue to be seemed beneath the microscope. These cells appeared pink due to dye.

### Chromophobe renal cell carcinoma

This includes few cases of RCCs. The cells of these cancers are also pale, like the clear cells, but are much larger and vary in other habits, as well.

### Collecting duct renal cell carcinoma

This subtype is very uncommon. The major characteristic is that the cancer cells have ability to form asymmetrical tubes.

### Unclassified renal cell carcinoma

In exceptional cases, renal cell cancers are categorized as "unclassified" since they do not fit into any of the previous groups or because of the presence of more than one type of cell.

## Stages of Kidney Cancer

**Figure 1:**
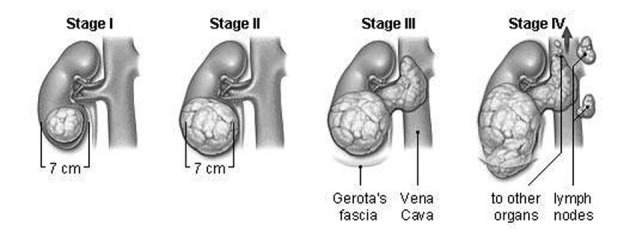
Stages of tumor growth in kidney.

On the basis of tumor size and its distribution to the other parts of body, kidney cancer can be separated into following four stages which might be helpful in the prediction of the line of treatment. The stages of tumor growth in kidney are given in Fig. 1.

### Stage I

The tumor is small (less than 7 centimeters) and has not spread outside the kidney.

### Stage II

The tumor is larger than 7 centimeters.

Patients with stage I and II RCC most often have their cancers surgically removed by either radical or partial nephrectomy. Additional (adjuvant) chemotherapy, radiation therapy, or immunotherapy after surgery for stage I or stage II RCC is not recommended [14].

### Stage III

The tumor has either: 1) started to develop elsewhere of the kidney, into the neighbouring fat tissue, or 2) spread to a close lymph node, or 3) spread to the chief blood vessels of the kidney.

Radical nephrectomy is the most common treatment for stage III RCC [14].

### Stage IV

The tumor has spread into more than one lymph node, or it has spread widely to other areas of the body, for example the lungs, bone, or brain.

Stage IV RCC has spread too far away from the kidney to be cured by surgery [14].

## Risk Factors of Kidney Cancer

The exact cause of kidney cancer is unknown. However, some epidemiologic data shows that age beyond 50 years, male gender, and end-stage renal disease are risk factors for developing kidney cancer. The common risk factors which have been predicted earlier are as follows:

### Lifestyle-related and job-related risk factors

#### Smoking

Smoking enhances chances of kidney cancer. The risk appears to be related to how much a person smokes and dives if discontinue smoking [15].

#### Obesity

An extremely overweight person has a higher danger of getting kidney cancer [16].

#### Job hazards

Many studies propose that exposure to certain chemicals on the job raises the risk of kidney cancer. Some of these are asbestos, cadmium, some herbicides, benzene, and organic solvents, mainly trichloroethylene [16].

### Inherited risk factors [17]

Rare innate condition may also be the reasons for development of kidney cancer. People with these conditions have a much higher risk for getting kidney cancer, but they report for only a small fraction of cases overall. Some rare inherited conditions are listed below. 1), Von Hippel-Lindau disease. 2), Hereditary papillary renal cell carcinoma. 3), Hereditary leiomyoma-renal cell carcinoma. 4), Birt-Hogg-Dube syndrome. 5), Hereditary renal oncocytoma.

### Other risk factors

#### Family history

Person with family history (especially a brother or sister) of kidney cancer has much chances of developing the disease.

#### High blood pressure

High blood pressure may also increase the risk of kidney cancer. People with high blood pressure are frequently treated with drugs, thus it quite tough to recommend that the reason behind the higher risk is due to the drugs, by the high blood pressure itself, or both.

#### Certain medicines

A previously accepted analgesic (called phenacetin) has been related to kidney cancer. However, this medicine was banned in the United States for over 20 years, and it no longer appears to be a major risk factor. Some drugs used to treat high blood pressure have also been linked to kidney cancer. It is unclear whether the higher risk is caused by the drugs or the high blood pressure.

#### Advanced kidney disease

People with advanced kidney disease who need to be on dialysis have a higher risk of kidney cancer. Dialysis is a treatment used to eliminate toxins from the body in people whose kidneys are not functioning.

#### Gender

Kidney cancer is found about twice as often in men as in women. However, the reasons for this are not apparent.

#### Race

African Americans have a slightly higher rate of renal cell cancer than whites. The reasons for this are also not clear.

Most probable, kidney cancer is caused by a combination of sporadic genetic events, enironmental exposures, and patient factors [18, 19].

## Signs and Symptoms of Kidney Cancer

People with kidney cancer may experience: 1) Blood in the urine. 2) Pain or pressure in the side or back. 3) A lump in the side or back. 4) Ankle and leg swelling. 5) High blood pressure. 6) Anaemia (low levels of red blood cells). 7) Weakness and fatigue. 8) Loss of appetite. 9) Weight loss.

## Diagnosis of Kidney Cancer [20]

On the basis of symptoms, the expert may carry out one or more of the following procedures:

### Physical exam

The doctor confirms common signs of health and tests for fever and high blood pressure. The doctor also senses the abdomen and site for tumors.

### Urine tests

Urine is analysed for blood and other signs of disease.

### Blood tests

This is done to observe the kidney functioning. An elevated level of several substances, such as creatinine signifies the improper working of kidneys.

### Intravenous pyelogram (IVP)

In this method dye is injected into a vein in the arm. The dye travels through the body and accumulates in the kidneys. A sequence of x-rays then follows the dye as it travels through the kidneys to the ureters and bladder. The x-rays confirms the presence of kidney tumor or any other problems.

### CT scan

With the help of an x-ray machine connected to computer a series of complete pictures of the kidneys could be captured. Injected dye to the patient may clearly displayed pictures of kidney which can be utilized for prediction of kidney cancer. Abdominal and chest CT provides information on primary tumour extension, morphology of the contralateral kidney, and evaluation of metastases [21].

### Ultrasound test

An ultrasound is a test that uses high frequency sound waves to produce an image of the body part being scanned. The waves bounce off the kidneys, and a computer uses the echoes to create a picture called a sonogram. A solid tumor or cyst shows up on a sonogram.

### Biopsy

A biopsy is the removal of tissue from suspected body part to look for cancer cells. A thin needle inserted through the skin into the kidney to remove a small portion of tissue. A pathologist uses a microscope to look for cancer cells in the tissue. Renal tumour biopsy is increasingly being used in diagnosis and is constantly designated previous to ablative and systemic therapy without earlier histopathology and in surveillance strategies to stratify follow-up [22].

## Treatment Options

### Surgical therapy

Surgery is the major treatment for kidney cancer that has not yet spread. The commonly used surgical method is laparoscopy, in this method surgeon makes several small incisions in the abdomen to introduce a tiny light, a camera, and instruments used to observe and eradicate the tumor. This type of surgery has been revealed to be just as efficient as conventional surgery and easier to recover from. However, a number of huge studies of metastatic kidney cancer have shown that people whose tumors are removed live longer than those whose tumors are not removed. An operation to remove the kidney is called a nephrectomy. As per the physicians each operation can be explained and discussed which is most appropriate for the patient:

#### Radical nephrectomy

It is the most commonly used method. This method includes the complete removal of whole kidney along with the adrenal glands and some periphery tissue of kidney. Some lymph nodes in the area also may be removed. Long-term outcome data indicate that laparoscopic radical nephrectomy has equivalent cancer-free survival rates to those of open radical nephrectomy [23].

#### Simple nephrectomy

This method includes the removal of just kidney itself and can be applied for the patient with stage I kidney cancer.

#### Partial nephrectomy

It involves the removal of only the part of the kidney that contains the tumor. This type of surgery may be used when the patient has only one kidney, or when the cancer affects both kidneys. Furthermore, a patient with a small kidney tumor (less than 4 centimeters or 3/4 of an inch) may have this type of surgery.

### Non-surgical therapies

#### Immunotherapy

It includes the medications which are used to increase the body’s natural ability to fight cancer. Two such drugs, interleukin-2 (Proleukin) and interferon alfa (Intron A, Roferon-A), can cause the shrinkage of some kidney tumors about more than half. Nevertheless, immunotherapy works in only 10 percent to 15 percent of patients. Still, in about 5 percent to 10 percent kidney cancer patients, interleukin-2 can direct to a long-term reduction of metastatic cancer. In several cases the tumors even vanish, and survivals of patients have increased up to 20 years after their kidney cancer diagnosis. Researchers are trying to discover ways to recognize those patients which are most liable to get benefit from immunotherapy. Immunotherapy is often combined with newer medications called targeted treatments.

#### Targeted therapies

Recent advances in molecular biology have led to the development of novel agents for the treatment [24]. In contrast with chemotherapy, targeted treatments attack specific molecules and cell mechanisms which are required for carcinogenesis and tumor growth. This specific targeting helps to spare healthy tissues and reduce side effects. Targeted cancer therapies may be more effective than current treatments and less injurious to normal cells. Research has revealed that addition of these targeted treatments to immunotherapy, or using them as a substitute of immunotherapy, nearly doubles the time duration so as to stop cancer growth.

##### 1) Sorafenib (Nexavar)

Sorafenib was approved by the U.S. Food and Drug Administration (FDA) in 2005 to treat metastatic kidney cancer. It is an oral multiple tyrosine kinase (TK) inhibitor, was in a phase 3 trial compared with placebo in patients in whom previous immunotherapy unsuccessful or who were unhealthy for immunotherapy. The trial reported that as compared with placebo, treatment with sorafenib prolongs progression-free survival in patients with advanced clear-cell renal-cell carcinoma in whom previous therapy has failed; however, treatment is associated with increased toxic effects [8]. Survival seems to improve in patients who crossed over from placebo to sorafenib treatment. It can be taken in pill form.

Sorafenib has been shown to shrink kidney tumors in many people who have previously tried other treatments that didn’t work. Sorafenib works by stopping vascular endothelial growth factor (VEGF) and platelet derived growth factor (PDGF) from stimulating the growth of new blood vessels in tumors. Because normal tissues have an established blood supply, they are not affected by the medication. Common side effects of the medication, such as loose stools, are generally easy to treat.

Recent findings showed that the sequence of first-line sorafenib followed by second line sunitinib resulted in a longer duration of response than did the oppo­site sequence. Sorafenib efficacy in first-line therapy can be potentiated by co-administration of low-dose interferon [25].

##### 2) Sunitinib (Sutent)

In 2006, the FDA approved sunitinib for treatment of metastatic kidney cancer. Like sorafenib, sunitinib is a pill that can be taken by mouth. It is taken once a day for four weeks followed by a two-week break, then another four week cycle.

Sunitinib is an orally administered multitargeted tyrosine kinase inhibitor of vascular endothelial and platelet-derived growth factor receptors [26]. In a phase 3 first-line trial comparing sunitinib with IFN-a, sunitinib achieved a longer progression-free survival than IFN-a while this advantage was limited to low- and intermediate-risk patients [27]. The overall survival highlights a superior prognosis in patients with RCC in the period of targeted therapy.

Since it is so efficient, sunitinib is often used as a first-line treatment for metastatic kidney cancer. Researchers also have shown that sunitinib can shrink kidney tumors in many people who have already tried other treatments that did not work. The side effects of sunitinib include fatigue, mouth pain, hand and foot pain, diarrhea, and high blood pressure.

##### 3) Temsirolimus (Torisel)

In May 2007, the FDA approved temsirolimus for the treatment of metastatic kidney cancer. Temsirolimus is a specific mammalian target of rapamycin (mTOR) inhibitor [9]. Patients with high-risk mRCC were randomised to receive first-line treatment with temsirolimus or IFN-a monotherapy or temsirolimus with IFN-a. In patients treated with combined temsirolimus plus IFN-a, overall survival was not significantly improved [9].

The side effects of temsirolimus are similar to those of the other targeted treatments used for metastatic kidney cancer. They include rash, mouth sores, fatigue, nausea, and sometimes low blood cell counts.

##### 4) Pazopanib (Votrient)

It is an oral angiogenesis inhibitor targeting vascular endothelial growth factor receptor, platelet-derived growth factor receptor, and c-Kit [28, 29]. In a current prospective randomised trial of pazopanib versus placebo in treatment-naive or cytokine-treated mRCC patients, Pazopanib demonstrated significant improvement in progression-free survival and tumor response compared with placebo in treatment-naive and cytokine-pretreated patients with advanced and/or metastatic RCC [30].

The most common adverse events of this trial were diarrhea, hypertension, hair color change, nausea, anorexia, and vomiting.

##### 5) Bevacizumab (Avastin)

It is a recombinant humanized monoclonal IgG1 antibody that binds to and inhibits the biologic activity of human vascular endothelial growth factor (VEGF). In a phase 3 trial, bevacizumab plus IFN-a was compared with IFN-a monotherapy [31]. The median overall response was 31% versus 13% for IFN-a only (p < 0.0001). Median progression-free survival increased significantly for bevacizumab plus IFN-a (p < 0.0001) but only in low-risk and intermediate-risk patients. No benefit was observed in high-risk patients. The common side-effect associated with bevacizumab is heart problems, high blood pressure, and nervous system and vision disturbances [11].

##### 6) Everolimus (Afinitor)

Everolimus is an orally administered inhibitor of the mammalian target of rapamycin (mTOR), a therapeutic target for metastatic renal cell carcinoma. A phase 3 study in 2008 compared everolimus versus placebo in mRCC patients also treated with finest supportive care and who had failed previous targeting treatment. Median progression-free survival was 4 mo with everolimus versus 1.9 mo with placebo (p < 0.001) [12], Which confirm that treatment with everolimus prolongs progression-free survival relative to placebo in patients with metastatic renal cell carcinoma that had progressed on other targeted therapies.

The common side-effects include cough, shortness of breath, diarrhea, rash, dry skin and itching, nausea and vomiting, fever and loss of appetite.

##### 7) Axitinib

It is an inhibitor of the receptor tyrosine kinases (RTKs) VEGFR1, VEGFR2 (vascular endothelial growth factor receptor) and PDGFR (platelet-derived growth factor receptor) with an acceptable side-effect profile consisting primarily of hypertension and stomatitis [32]. A phase II study of axitinib in 52 patients with metastatic RCC, all of whom had unsuccessful at least one previous cytokine-based therapy has just reported [33]. A response rate of 44% was seen with two (4%) complete responses and 21 (40%) partial responses. Median time to progression was 15.7 months and median overall survival was 29.9 months. Treatment-related adverse events included nausea, fatigue, diarrhoea, hoarseness and anorexia or weight loss.

The targeted agents used in the treatment of RCC are well accepted but the reason behind their toxicity is unidentified. This is primarily necessary if they are revealed to have a function as adjuvant therapy. Sunitinib and sorafenib both, have been reported for causing thyroid dysfunction [34, 35], moreover left ventricular dysfunction has been observed with sunitinib [7]. It is essential that these toxicities are recognised, examined and treated accordingly.

#### Chemotherapy

Several chemotherapeutic agents are being investigated for destruction of cancerous cells of kidney and other cancer of body. The specific type of chemotherapy depends on the site of metastases, type and grade of tumor, and physical condition of the patient. But unfortunately, these drugs are proven resistant to renal cell carcinoma [36]. However, several researches for the development of more effective drugs are under progress, surgery and targeted therapies stay the gold standard treatment for kidney cancer. A comprehensive review of 83 trials involving more than 4,000 patients reported a chemotherapy response rate of 6% [37].

Previously, patients have revealed some response to therapy with the single agent’s floxuridine, 5-fluorouracil, and vinblastine. Floxuridine and 5-fluorouracil are antimetabolites that work by inhibiting thymidylate synthase, a pivotal enzyme that catalyzes the de novo production of thymidylate and thymidine nucleotides that are necessary for DNA synthesis. Vinblastine binds to tubulin, causing inhibition of the mitosis phase of the cell cycle. A comprehensive review reported overall response rates of 43% or lower in patients receiving floxuridine, an overall response rate of 10% in patients taking 5-fluorouracil, and overall response rates of 7% or lower in patients receiving vinblastine [38].

Gemcitabine has some dated phase II evidence in a phase II trial and the response rates were quite low. There are more recent studies in combination with other drugs. A small clinical trial evaluated the combination of Gemzar®, alfa interferon and Proleukin® in 16 patients with metastatic renal cell cancer [39]. They found that Gemcitabine does not appear to be very active by itself in kidney cancer but may turn out to be effective in combinations.

#### Radiation Therapy [40]

Radiation is mostly used as a secondary treatment for kidney cancer that has metastasized to the bone, brain or spine. It may be used to control symptoms for example relief from pain. There are many type of radiation therapy which works on the similar fundamental theory of using high-energy radiation to kill cancer cells or slow their rate of growth. High-energy radiation is delivered to kidney cancer cells from outside the body as a tightly focused beam generated by computer planning. Radiation therapy is a “localized” treatment, targeted as accurately as possible at a specific area or tumor. Radiation damages the DNA molecules inside the cancer cell, thus preventing their growth. Unfortunately, radiation may also damage healthy, normal tissue. Side effects of radiation therapy occur in the area treated, referred to as the “radiation field.” These side effects are temporary and vary depending on the area of the body being treated. One of the most common side effects is dry, irritated (reddened) and sensitive skin.

Radiosurgery is non-surgical method that permits treatment of cancer that has metastasized to the brain. This allows for a more precise and concentrated treatment than other types of radiation. The highly-specialized radiation, called gamma-knife surgery, has been outstandingly successful in controlling brain metastases from kidney cancer, allowing patients improved disease control to receive targeted therapy.

#### Innovative Therapies [41]

##### 1) Cryoablation

Cryoablation destroys the kidney tumor by rapidly freezing it to a temperature of -40 °C, and then quickly melting it. The process disrupts the cells’ membranes and inner machinery as starving them of blood flow, oxygen and water. This cycle is repeated a second time to demolish any residual cellular processes. This practice is typically executed laparoscopically and is best for patients who are not good candidates for surgery due to age or other medical problems.

##### 2) Radiofrequency tumor ablation (RFA)

RFA employs electric currents to heat the tumor, causing direct cell death, injury and destruction to its blood supply. Most RFA procedures can be performed percutaneously (through the skin) under radiographic supervision. This procedure is also best for patients who are not good candidates for surgery due to age or other medical problems.

## Conclusion

The treatment of patients with advanced RCC has developed rapidly over the last decade. Several agents are now approved for use in many other countries, and patient viewpoint is significantly brighter. Making progress in the area of immunotherapy, targeted therapy and chemotherapy is necessary in order to significantly reduce morbidity and mortality related to advance RCC. The development of these agents with improved efficacy, of which, the kinase inhibitors have demonstrated the most significant activity. Progressively more oncologists have choices concerning treatment selections for patients with renal cell carcinoma. Studies to date raise many queries with regard to scheduling, dose, duration, potential combinations and toxicity of treatment. A profound perceptive of the bond between the signalling pathways motivating tumor growth and their inhibition is needed in order to optimise the use of these agents and should recommend strategies to hinder or stop resistance and identify suitable therapeutic combinations. Current era is hopeful with regard to treatment for renal cell carcinoma with growing numbers of active agents. To understand these hopes, this is the major challenge to counterpart these agents with the biology of the tumors and their hosts.
